# Supplementary material for: Anchovy boom and bust linked to trophic shifts in larval diet
Source: Nat Commun. 2023 Dec 5;14:7412. doi: 10.1038/s41467-023-42966-0 (PMC10698165; doi:10.1038/s41467-023-42966-0)
Supplement: Supplementary file 1 — Supplementary Information [file 41467_2023_42966_MOESM1_ESM.pdf]

Supplementary Information:

Anchovy boom and bust linked to trophic shifts in larval diet

Rasmus Swalethorp, Michael R. Landry, Brice X. Semmens, Mark D. Ohman, Lihini Aluwihare, Dereka Chargualaf, Andrew R. Thompson

Supplementary Figures

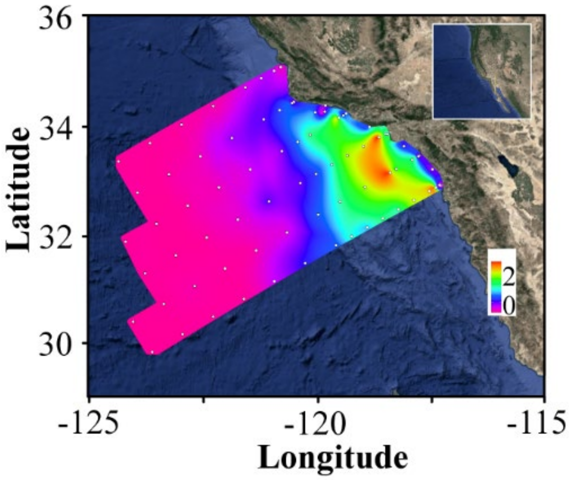

Supplementary Figure 1. CalCOFI stations and distributions of anchovy larvae in the California Current Ecosystem. Mean distribution of the larvae from the central anchovy stock over the CalCOFI time series. Colors depict standardized z-scores based on station means from 1951-2015. Source data are provided as a Source Data file.

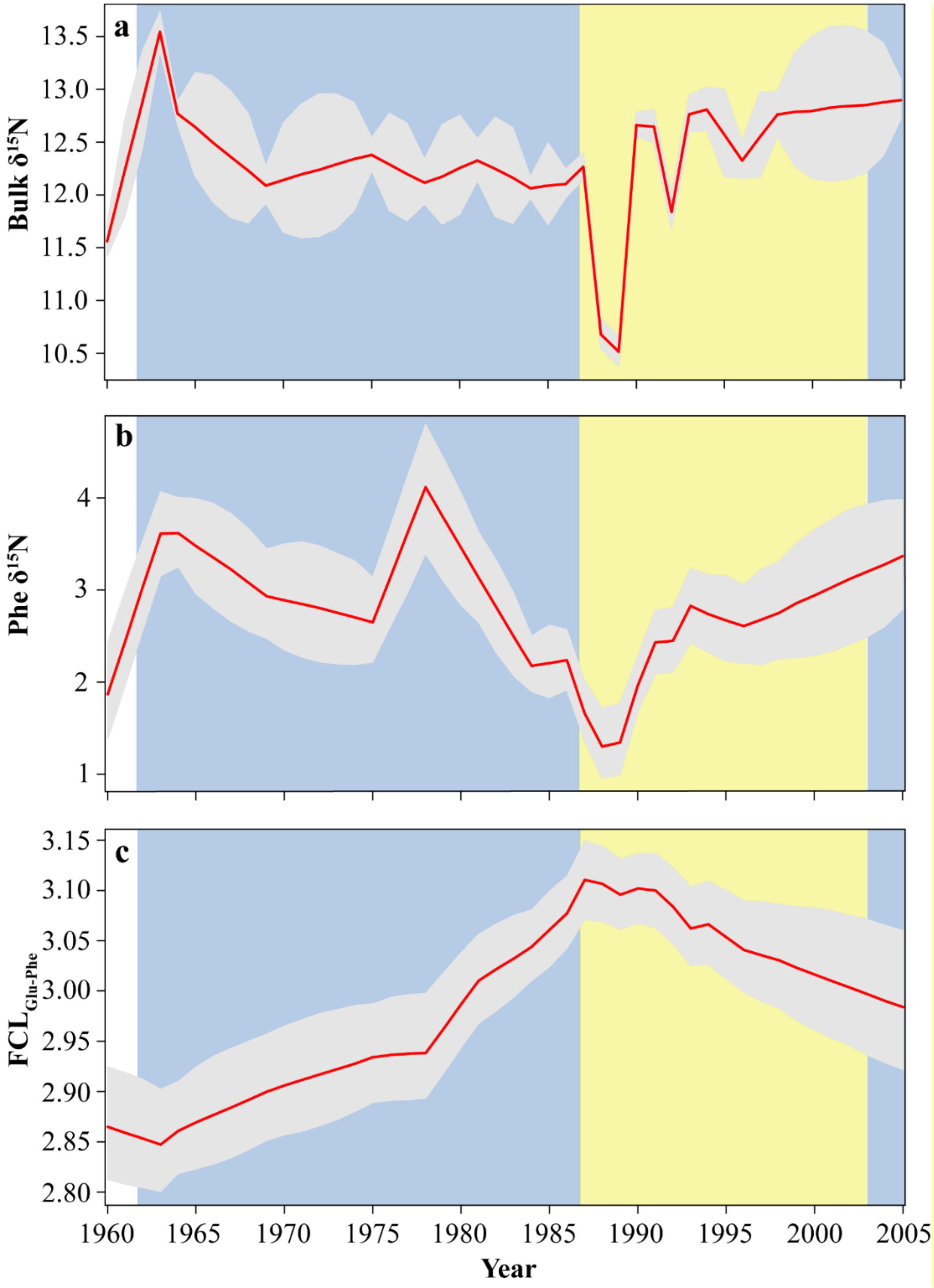

Supplementary Figure 2. 45-year time series of larval bulk and Phe $\delta^{15}N$ and food chain length (FCL) dynamics. Bayesian state space model estimated (A) anchovy larvae bulk $\delta^{15}N$ (n = 207), (B) the source AA Phe $\delta^{15}N$, and (C) $FCL_{Glu\text{-}Phe}$ calculated from one trophic and one source AA for larval anchovy (n = 199), Error bands denote the 95% credible interval. Source data are provided as a Source Data file.

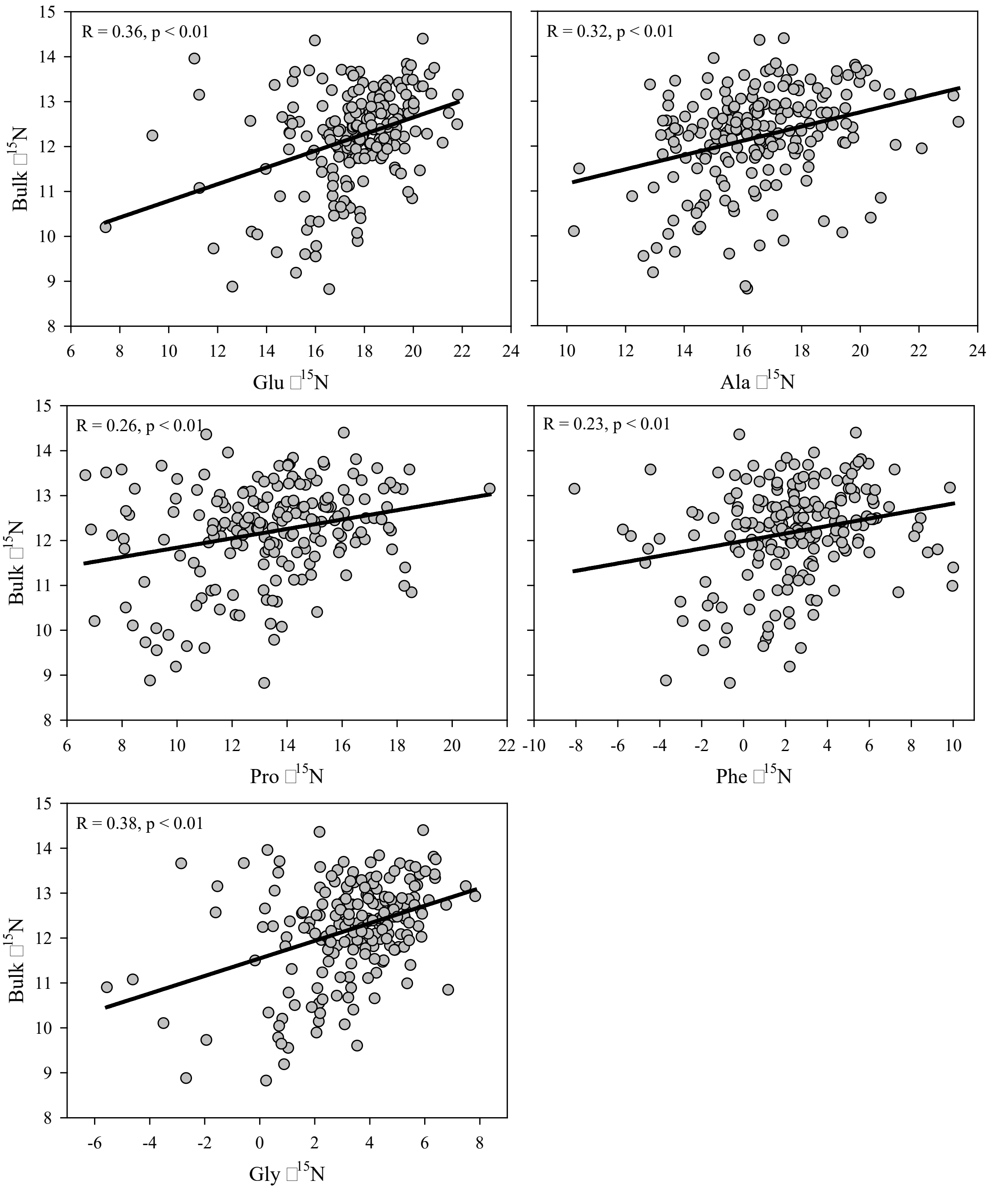

Supplementary Figure 3. Correlations of $\delta^{15}N_{Bulk}$ and $\delta^{15}N_{AA}$ values in larval anchovy. Individual panels show relationships between bulk δ^{15}N values and individual source AAs (Phe, Gly) and trophic (Glu, Ala, Pro) AAs along with the Pearson product moment correlation coefficient (R) and level of significance (n=199). The correlations indicate that bulk δ^{15}N integrates signatures of both the base of the food chain and at the level of the consumed prey. Source data are provided as a Source Data file.

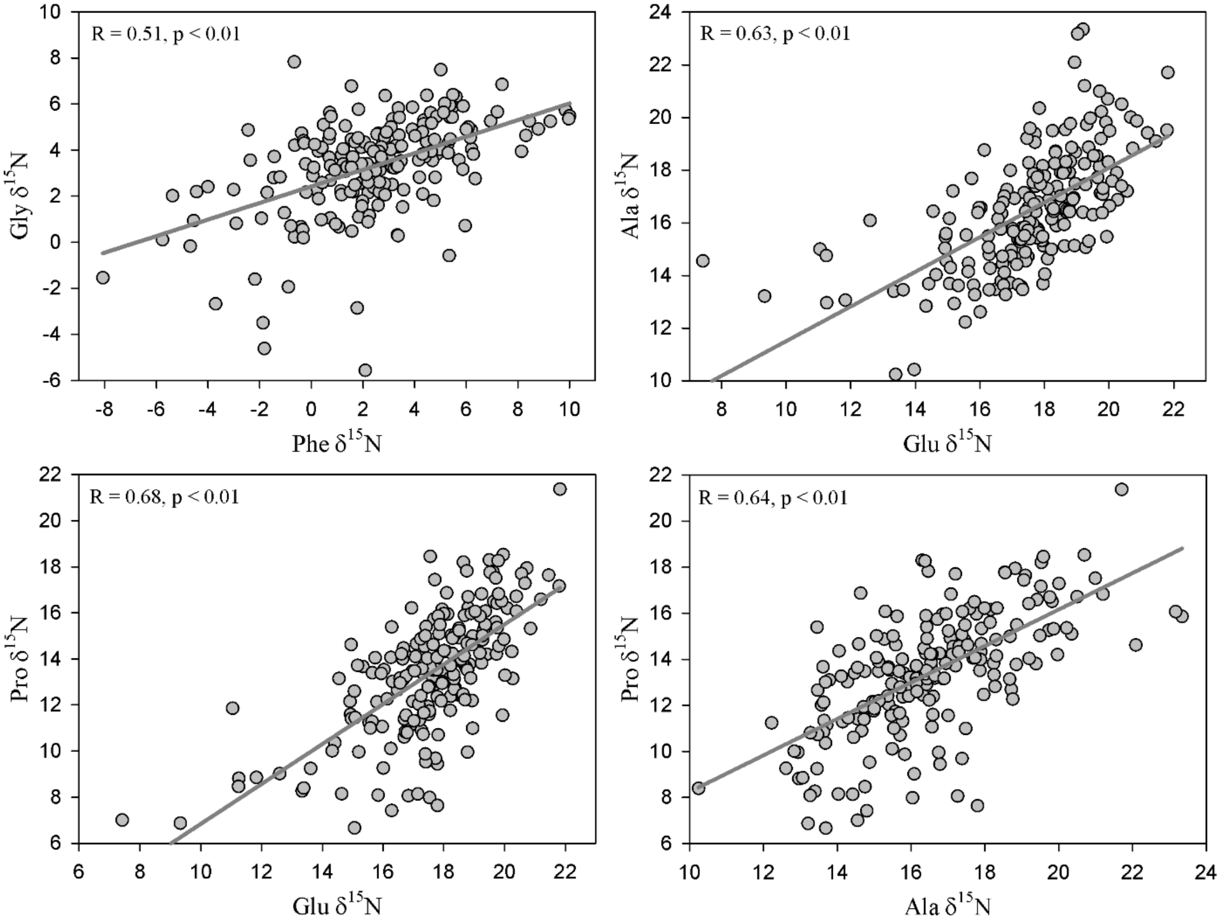

Supplementary Figure 4. Correlations of $\delta^{15}N_{AA}$ values in larval anchovy. Panels show relationships between $\delta^{15}N$ values for individual source (Phe, Gly) and trophic (Glu, Ala, Pro) AAs along with the Pearson product moment correlation coefficient (R) and level of significance (n=199). Source data are provided as a Source Data file.

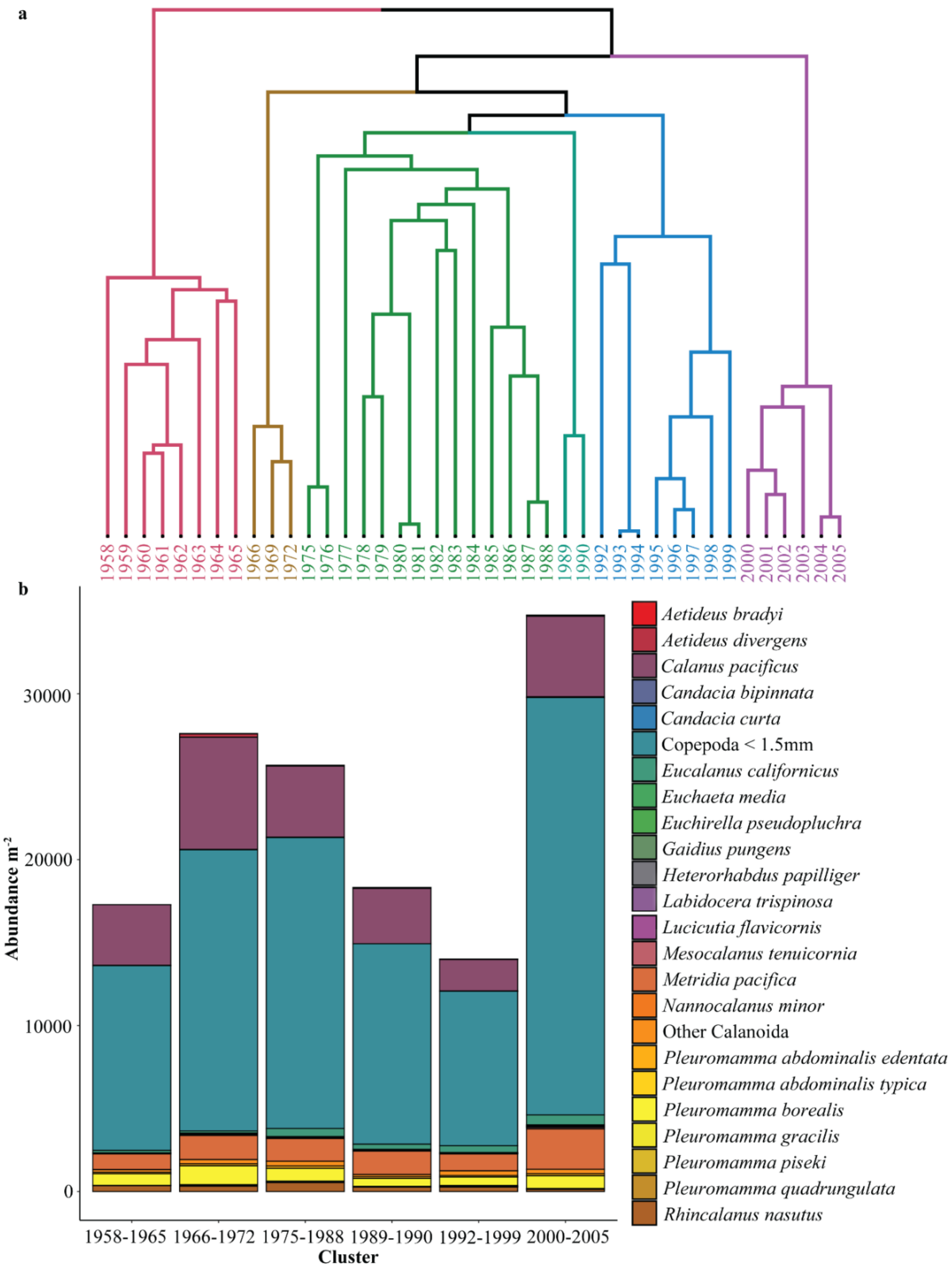

Supplementary Figure 5. Copepod community analysis from 1958 to 2005. (A) Chronological clustering dendrogram showing six distinct deep breaks in year clusters of communities and (B) bar plot showing the average community composition of each year cluster. Source data are provided as a Source Data file.

Larval variable	Body or Environmental variable	Test	Model
Bulk $\delta^{15}N$<br>Phe $\delta^{15}N$<br>$FCL_{Trp\text{-}Scr}$<br>$FCL_{Glu\text{-}Phe}$	Standard length<br>Body height at anal fin	Larvae specific	GLM
Bulk $\delta^{15}N$<br>Phe $\delta^{15}N$<br>$FCL_{Trp\text{-}Scr}$<br>$FCL_{Glu\text{-}Phe}$	Distance to shore<br>Dynamic height<br>Sea surface temperature<br>Temperature 0-30 m depth average<br>Salinity 0-30 m depth average<br>Oxygen 0-30 m depth average<br>Nitrate 0-30 m depth average<br>Chlorophyll *a* 0-30 m depth average<br>Zooplankton biovolume	Sampling station specific	LMM, Correlation
Bulk $\delta^{15}N$<br>Phe $\delta^{15}N$<br>$FCL_{Trp\text{-}Scr}$*<br>$FCL_{Glu\text{-}Phe}$*<br>ETE*<br>Larval size ratio*	Zooplankton biovolume*<br>Copepod abundance<br>Copepod abundance (<1.5 mm)<br>*Calanus pacificus* abundance*<br>Crustacea abundance<br>Crustacea abundance (<1.5 mm)<br>All none glatinous zooplankton abundance<br>All none glatinous zooplankton abundance (<1.5 mm)	Cruise specific	Cross correlate
Bulk $\delta^{15}N$<br>Phe $\delta^{15}N$<br>$FCL_{Trp\text{-}Scr}$<br>$FCL_{Glu\text{-}Phe}$<br>ETE<br>Larval size ratio<br>SSB	Pacific Decadal Oscillation (PDO)<br>NPGO<br>MEI<br>Bakun upwelling index<br>Wind stress curl<br>Ekman North transport<br>Ekman East transport<br>Ekman offshore transport	Cruise specific	Cross correlate

Supplementary Table 1. List of larval body metrics and environmental variables tested (Energy transfer efficiency; ETE). * indicates strong correlation between larval and environmental variables.

Supplementary Tables

Year	No. of stations sampled	No. of Bulk SIA samples	No. of CSIA-AA samples
1960	4	11	11
1963	5	7	7
1964	9	20	17
1969	3	8	7
1975	7	10	9
1978	3	5	5
1981	4	6	6
1984	12	22	22
1986	10	12	12
1987	9	15	15
1988	8	12	11
1989	6	12	11
1990	16	18	18
1991	2	9	9
1992	6	8	8
1993	4	7	7
1994	4	5	5
1996	5	8	8
1998	4	5	4
2005	4	7	7

Supplementary Table 2. Number of individual larvae per sampling year used for Bulk SIA and CSIA-AA analysis.

Year	Bulk $\delta^{15}N$	Glu $\delta^{15}N$	Ala $\delta^{15}N$	Pro $\delta^{15}N$	Phe $\delta^{15}N$	Gly $\delta^{15}N$	$FCL_{Glu-Phe}$	$FCL_{Trp-Scr}$
1960	11.5 ± 1.5	15.4 ± 3.4	15.3 ± 2.6	11.7 ± 3.0	0.9 ± 2.6	2.9 ± 2.2	2.9 ± 0.4	2.6 ± 0.2
1963	13.7 ± 0.3	17.9 ± 3.3	17.9 ± 2.0	15.1 ± 2.4	4.9 ± 1.4	4.2 ± 3.0	2.6 ± 0.5	2.6 ± 0.2
1964	12.7 ± 0.6	18.1 ± 2.1	17.3 ± 2.1	14.6 ± 2.3	3.9 ± 4.0	4.0 ± 1.8	2.9 ± 0.4	2.6 ± 0.4
1969	12.1 ± 0.3	17.2 ± 1.4	15.6 ± 2.3	13.3 ± 3.0	2.7 ± 3.5	3.6 ± 1.5	2.9 ± 0.5	2.5 ± 0.4
1975	12.4 ± 0.4	16.5 ± 3.1	16.5 ± 3.1	13.3 ± 2.8	1.8 ± 3.5	3.9 ± 2.0	3.0 ± 0.4	2.7 ± 0.3
1978	12.1 ± 0.7	19.5 ± 1.8	17.5 ± 2.4	17.3 ± 2.8	6.7 ± 2.8	5.0 ± 1.5	2.6 ± 0.5	2.3 ± 0.4
1981	12.4 ± 0.5	19.1 ± 0.5	17.5 ± 2.1	15.3 ± 1.8	3.3 ± 5.0	4.8 ± 1.5	3.1 ± 0.8	2.7 ± 0.7
1984	12.1 ± 0.6	17.1 ± 1.3	15.1 ± 1.5	12.5 ± 2.4	1.9 ± 2.5	3.1 ± 1.3	3.0 ± 0.4	2.5 ± 0.3
1986	12.1 ± 0.4	17.9 ± 1.4	16.1 ± 1.8	13.3 ± 1.7	3.0 ± 2.4	3.9 ± 0.8	3.0 ± 0.3	2.5 ± 0.2
1987	12.4 ± 0.9	18.1 ± 1.6	17.1 ± 1.7	12.3 ± 2.8	1.4 ± 2.9	3.5 ± 2.1	3.3 ± 0.4	2.8 ± 0.2
1988	10.6 ± 1.0	16.5 ± 1.2	15.5 ± 2.1	12.6 ± 2.0	0.6 ± 2.0	1.7 ± 1.2	3.2 ± 0.4	2.8 ± 0.3
1989	10.3 ± 0.9	15.5 ± 2.3	14.7 ± 2.5	10.6 ± 1.7	0.5 ± 2.0	-0.5 ± 3.0	3.0 ± 0.2	2.8 ± 0.4
1990	12.8 ± 0.7	17.9 ± 1.5	16.8 ± 1.8	12.5 ± 2.4	2.1 ± 2.1	3.0 ± 1.9	3.1 ± 0.4	2.7 ± 0.3
1991	12.7 ± 0.4	19.4 ± 1.3	18.1 ± 1.3	14.7 ± 1.8	3.2 ± 1.7	4.0 ± 1.2	3.2 ± 0.2	2.8 ± 0.2
1992	11.6 ± 1.2	17.6 ± 1.4	17.0 ± 2.0	13.6 ± 1.9	1.9 ± 1.7	3.2 ± 0.9	3.1 ± 0.2	2.8 ± 0.3
1993	12.9 ± 1.2	17.9 ± 1.7	18.0 ± 1.9	15.2 ± 2.8	3.8 ± 2.6	4.0 ± 1.6	2.8 ± 0.3	2.7 ± 0.2
1994	12.9 ± 0.3	19.4 ± 0.8	17.0 ± 1.7	14.5 ± 2.4	2.8 ± 4.4	3.9 ± 2.0	3.3 ± 0.6	2.7 ± 0.3
1996	12.3 ± 0.6	17.2 ± 1.8	15.9 ± 1.3	13.8 ± 2.9	2.3 ± 1.5	3.1 ± 1.8	3.0 ± 0.3	2.6 ± 0.1
1998	12.8 ± 0.7	17.9 ± 1.6	17.6 ± 1.7	14.1 ± 1.8	2.7 ± 2.1	4.1 ± 1.6	3.0 ± 0.4	2.7 ± 0.2
2005	12.9 ± 0.4	18.0 ± 1.2	17.2 ± 1.8	15.1 ± 2.3	3.7 ± 1.5	4.5 ± 1.6	2.9 ± 0.3	2.6 ± 0.2

Supplementary Table 3. Larval bulk and amino acid specific δ15N values and FCL estimates averaged for each year ± Sd.
