# Supplementary material for: Anchovy boom and bust linked to trophic shifts in larval diet
Source: Nat Commun. 2023 Dec 5;14:7412. doi: 10.1038/s41467-023-42966-0 (PMC10698165; doi:10.1038/s41467-023-42966-0)
Supplement: Supplementary file 3 — Reporting Summary [file 41467_2023_42966_MOESM3_ESM.pdf]

nature portfolio

Corresponding author(s): Rasmus Swalethorp

Last updated by author(s): Jul 7, 2023

Reporting Summary

Nature Portfolio wishes to improve the reproducibility of the work that we publish. This form provides structure for consistency and transparency in reporting. For further information on Nature Portfolio policies, see our Editorial Policies and the Editorial Policy Checklist.

Statistics

For all statistical analyses, confirm that the following items are present in the figure legend, table legend, main text, or Methods section.

n/a	Confirmed	
☐	☒	The exact sample size (*n*) for each experimental group/condition, given as a discrete number and unit of measurement
☐	☒	A statement on whether measurements were taken from distinct samples or whether the same sample was measured repeatedly
☒	☐	The statistical test(s) used AND whether they are one- or two-sided<br>*Only common tests should be described solely by name; describe more complex techniques in the Methods section.*
☐	☒	A description of all covariates tested
☐	☒	A description of any assumptions or corrections, such as tests of normality and adjustment for multiple comparisons
☐	☒	A full description of the statistical parameters including central tendency (e.g. means) or other basic estimates (e.g. regression coefficient) AND variation (e.g. standard deviation) or associated estimates of uncertainty (e.g. confidence intervals)
☒	☐	For null hypothesis testing, the test statistic (e.g. *F*, *t*, *r*) with confidence intervals, effect sizes, degrees of freedom and *P* value noted<br>*Give P values as exact values whenever suitable.*
☐	☒	For Bayesian analysis, information on the choice of priors and Markov chain Monte Carlo settings
☒	☐	For hierarchical and complex designs, identification of the appropriate level for tests and full reporting of outcomes
☐	☒	Estimates of effect sizes (e.g. Cohen's *d*, Pearson's *r*), indicating how they were calculated

Our web collection on statistics for biologists contains articles on many of the points above.

Software and code

Policy information about availability of computer code

Data collection	No software used
Data analysis	Microsoft Excel, SigmaPlot v. 12.0 software, R v. 4.1.3, R kriging package, R Vegan package, R CODA package, R rioja package, R ggplot2 package, R package R2jags, R package devEMF. https://github.com/BriceSemmens/ichthyoplankton_TS

For manuscripts utilizing custom algorithms or software that are central to the research but not yet described in published literature, software must be made available to editors and reviewers. We strongly encourage code deposition in a community repository (e.g. GitHub). See the Nature Portfolio guidelines for submitting code & software for further information.

Data

Policy information about availability of data

All manuscripts must include a data availability statement. This statement should provide the following information, where applicable:

- Accession codes, unique identifiers, or web links for publicly available datasets
- A description of any restrictions on data availability
- For clinical datasets or third party data, please ensure that the statement adheres to our policy

Summary table of all stable isotope data is available in Supplementary Table 3. Source data are provided with this paper both as raw data and state space model output data. CalCOFI hydrographic datasets used in this study can be found at https://calcofi.org/data/oceanographic-data/bottle-database/, larval anchovy population data at https://coastwatch.pfeg.noaa.gov/erddap/tabledap/, zooplankton community data at https://oceaninformatics.ucsd.edu/zoodb/, Pacific Decadal Oscillation Index from http://research.jisao.washington.edu/data_sets/pdo/#data, Multivariate ENSO Index from https://www.psl.noaa.gov/enso/mei.old/, North

Pacific Gyre Oscillation index from http://www.o3d.org/npgo/, and wind driven upwelling index and horizontal Ekman transport indices from https://oceanview.pfeg.noaa.gov/products.

Research involving human participants, their data, or biological material

Policy information about studies with human participants or human data. See also policy information about sex, gender (identity/presentation), and sexual orientation and race, ethnicity and racism.

Reporting on sex and gender	No human research participants used.
Reporting on race, ethnicity, or other socially relevant groupings	No human research participants used.
Population characteristics	No human research participants used.
Recruitment	No human research participants used.
Ethics oversight	No human research participants used.

Note that full information on the approval of the study protocol must also be provided in the manuscript.

Field-specific reporting

Please select the one below that is the best fit for your research. If you are not sure, read the appropriate sections before making your selection.

☐ Life sciences ☐ Behavioural & social sciences ☒ Ecological, evolutionary & environmental sciences

For a reference copy of the document with all sections, see nature.com/documents/nr-reporting-summary-flat.pdf

Ecological, evolutionary & environmental sciences study design

All studies must disclose on these points even when the disclosure is negative.

Study description	This study investigates the food chain length (FCL) and size ratios of larval anchovy over a 45-year period. FCL was determined by compound specific isotopic analysis of amino acids (CSIA-AA) in individual spring caught larval anchovy and grouped for each year. Larval size ratios were calculated from larval catch data from within our study site and grouped for each year.
Research sample	This study uses existing Norther Anchovy (Engraulis mordax) central stock larval catch information and archived formalin preserved samples from the California Cooperative Oceanic Fisheries Investigations (CalCOFI) program. These are the only samples and data that exist that would allow historical analysis of larval FCL and demographic shifts.
Sampling strategy	In total, 207 anchovy larvae were analyzed for stable isotope analysis over 20 Springs between 1960 and 2005 (Extended Data Table 1). For years of high larval catches, we focused on ≥ 3 core stations (defined as stations with the highest abundances of larvae in the desired size range), assuming these to have been collected from the most optimal nursery habitats and therefore most likely to contribute to recruitment. For years of low larval catches, we included larvae from all stations, assuming these to be the only ones that could contribute to recruitment. Within years and sampling stations, we took as wide a selection of larval sizes as possible within the defined size ranges. We did not use any statistical method to determine sample size since for most years analyzed we exhausted all available archived larval sample material. We ensured analysis of a minimum or 4 replicate larvae per year.<br>Analyzed larvae were in the standard length (SL) range of 18-23 mm. By selecting these size ranges, we ensured that the larvae had grown to ≥ 10 times their initial weight at yolk sack absorption, diluting the maternal isotopic signal, but still leaving sufficient larvae material for stable isotopic analysis (SIA) from a reasonable number of sampling years from the CalCOFI time series.
Data collection	The only new data collection that is presented was that of 15N values from individual anchovy larvae. Bulk SIA samples were analyzed at the Scripps Institution of Oceanography Stable Isotope Facility (SIO). Samples were analyzed on a Costech ECS 4010 Elemental Analyzer coupled to a Thermo Finnigan Delta Plus XP Isotope Ratio Mass Spectrometer. Sample nitrogen (15N/14N) ratios were reported using the δ notation relative to atmospheric nitrogen (N2). Measured δ15N values were corrected for size effects and instrument drift using acetanilide standards (Baker AO68-03, Lot A15467).<br>Amino acid samples were analyzed at the Stable Isotope Laboratory facility at the University of California, Santa Cruz (UCSC-SIL). Samples were analyzed on a Nano-EA-IRMS system designed for small sample sizes. The automated system is composed of a Carlo Erba CHNS-O EA1108 Elemental Analyzer connected to a Thermo Finnigan Delta Plus XP Isotope Ratio Mass Spectrometer via a Thermo Finnigan Gasbench II with a nitrogen trapping system similar to the configuration of Polissar et al. 83. Measured δ15N values were corrected for size effects and instrument drift using Indiana University acetanilide, USGS41 Glu and Phe standards and correction protocols (see https://es.ucsc.edu/~silab)
Timing and spatial scale	We focused on spring season when Northern Anchovy mainly spawn in the period between March 1960 to April 2005 as the CalCOFI program conducted extensive sampling of larval anchovy, larval sorting and recording of sizes and abundances during this time. This period covers an extended period of high anchovy spawning stock biomass (SSB), an extended period of low SSB and a brief one year resurgence to high SSB. Larval anchovy were not collected every spring in this time period as for a period of time cruises only occurred every 3rd year, and larger larvae that could be analyzed for stable isotopes were only available in 20 years over the 45 year period (1960, 1963, 1964, 1969, 1975, 1978, 1981, 1984, 1986, 1987, 1988, 1989, 1990, 1991, 1992, 1993, 1994, 1996, 1998, 2005). Samples used in this study were collected core CalCOFI grid between and including lines 93.3 and 76.7, which is the part of the

	sampling grid that has always been sampled throughout the 75 years of the program. The grid covers an area between San Diego and just north of Point Conception from close to shore going 375 nautical miles offshore (https://calcofi.org/sampling-info/station-positions/). This area largely covers the range of the central stock of Northern Anchovy.
Data exclusions	No data was excluded.
Reproducibility	The reproducibility of the compound specific isotopic analysis method used, and the possible effects of formalin preservation was determined and published in another paper by Swalethorp et al. 2020. Assessment of the reproducibility of the FCL time series was not carried out due to limited number of larvae to sample from the historical archives, limited larval size allowing only one CSIA-AA analysis per larvae in most cases, but we did ensure that several replicate larvae were analyzed from each year (n = 4-22).
Randomization	Samples were randomized during CSIA-AA analysis.
Blinding	Samples for stable isotope analysis were not labeled with time and location information but only a numerical number and then randomized. This way the stable isotope analytical facilities used in this study could not tell when or where a sample was taken.

Did the study involve field work? ☐ Yes ☒ No

Reporting for specific materials, systems and methods

We require information from authors about some types of materials, experimental systems and methods used in many studies. Here, indicate whether each material, system or method listed is relevant to your study. If you are not sure if a list item applies to your research, read the appropriate section before selecting a response.

Materials & experimental systems

n/a	Involved in the study
☒	☐ Antibodies
☒	☐ Eukaryotic cell lines
☒	☐ Palaeontology and archaeology
☐	☒ Animals and other organisms
☒	☐ Clinical data
☒	☐ Dual use research of concern
☒	☐ Plants

Methods

n/a	Involved in the study
☒	☐ ChIP-seq
☒	☐ Flow cytometry
☒	☐ MRI-based neuroimaging

Animals and other research organisms

Policy information about studies involving animals; ARRIVE guidelines recommended for reporting animal research, and Sex and Gender in Research

Laboratory animals	Study did not involve laboratory animals.
Wild animals	Larval anchovy from the CalCOFI monitoring programs historical archives.
Reporting on sex	Sex not determined in larval anchovy.
Field-collected samples	Samples were dead and formalin fixed.
Ethics oversight	No ethical approval or guidance was required as this study was carried out using existing historical archive samples. No live anchovy were collected in this study.

Note that full information on the approval of the study protocol must also be provided in the manuscript.
